# Quantification of Wine Mixtures with an Electronic Nose and a Human Panel

**DOI:** 10.3389/fbioe.2018.00014

**Published:** 2018-02-12

**Authors:** Manuel Aleixandre, Juan M. Cabellos, Teresa Arroyo, M. C. Horrillo

**Affiliations:** ^1^SENSAVAN, Instituto de Tecnologías Físicas y de la Información (ITEFI-CSIC), Madrid, Spain; ^2^Departamento Investigación Agroalimentaria, Instituto Madrileño de Investigación y Desarrollo Rural, Agrario y Alimentario (IMIDRA), Madrid, Spain

**Keywords:** electronic nose, aroma quantification, gas sensor, wine mixtures, human panel

## Abstract

In this work, an electronic nose and a human panel were used for the quantification of wines formed by binary mixtures of four white grape varieties and two varieties of red wines at different percentages (from 0 to 100% in 10% steps for the electronic nose and from 0 to 100% in 25% steps for the human panel). The wines were prepared using the traditional method with commercial yeasts. Both techniques were able to quantify the mixtures tested, but it is important to note that the technology of the electronic nose is faster, simpler, and more objective than the human panel. In addition, better results of quantification were also obtained using the electronic nose.

## Introduction

Nowadays, more information is required on the food products that the citizen consumes and even more on those with high added value, such as wine. The labeling of wine products should mention certain characteristics of the product, such as the alcoholic strength and the presence of sulfites. In addition, there are specific provisions governing the labeling of the various wine products and containing mandatory and optional indications for the labeling of each category of products.

Therefore, there is a European Regulation, Regulation (EC) No 1493/1999, on the common organization of the market in wine. The rules of this regulation help consumers to better understand the specificities of wine products and guarantee to producers, the value of the quality of their products. The purpose of this regulation is to protect the interests of consumers and producers through the establishment of certain implementing provisions.

An important aspect would be to achieve an objective system that provides information on grape varieties introduced into a bottle, thereby defending criteria of authenticity and protection of quality of wines, at least for a given region, thus avoiding possible commercial wine fraud.

The wine control is carried out in cellars using traditional records referred to as “winery books.” They must contain the land of origin of the grapes, the tanks where the wine have been fermented, and the subsequent trajectory of racking, mixing, clarification, rest in barrels, and bottling up, in summary the complete traceability cycle from source. In this way, one should be able to quantify the varieties that make up the wine that reach the consumer. It is important to develop an objective system that provides information on grape varieties introduced into a bottle, thereby defending criteria of authenticity and protection of quality of wines, at least for a given region, avoiding possible commercial wine fraud.

The tradition of wine blending began centuries ago. Blends are produced by mixing wine varieties in different proportions. Blending is mainly used to increase the complexity of the wine in order to enhance its organoleptic properties. Several studies have focused on the sensory and chemical properties of wine blends in comparison to their base wines, and in addition they are referred to as red wines (Hopfer et al., 2012; Hjelmeland et al., 2013).

Mixtures of different grape types are realized to obtain a wine with superior characteristics in terms of complexity, improvement of aroma, taste, and texture. In fact, the end result is a wine that meets the specific characteristics that each type of grape provides to create a better final product. They can be two or more types of red grapes, or two or more types of white grapes. It is very rare to see mixtures of red and white grapes. On the other hand, these mixtures of varieties can lead to erroneous labeling and even to fraud, so low quality wines can be mixed with other high quality wines, in unstated proportions and sold as very expensive wines, as can be seen in the following reference (Georges-Duboeufs, 2006).

Therefore, there is a great need to develop techniques to quantify mixtures of wines, with the aim of improving their quality control and authentication. Besides, it is important that these techniques are objective, simple operating, less-expensive, and fast and reversible in their response, and they can measure in real-time and on-line. These requirements are fulfilled by the electronic nose technique, which has been widely used for different wine applications (Gardner and Bartlett, 1999; Di Natale et al., 2004; Gil-Sánchez et al., 2011; Wei et al., 2014; Rodríguez-Méndez et al., 2016). Even though the human panel technique is very much used in the wine world, it is limited, mainly due to its subjectivity and fatigue over long periods. The electronic noses developed in our laboratory, based mainly on resistive sensors, have been used in many wine applications (Horrillo et al., 2007; Aleixandre et al., 2008; Lozano et al., 2008, 2015; Arroyo et al., 2009; Santos et al., 2010). In the last published work, it was possible to differentiate samples of the same grape variety that were made using grapes from the same vineyard, but harvested at different times of ripeness (Aleixandre et al., 2015).

In the present investigation, the application of a sensor array to quantify four wine mixtures prepared with different grape varieties, at different proportions, demonstrated good results. In addition, the results obtained are compared to those ones achieved using the human panel technique. The wines were made in the Experimental Cellar “El Encín” (Alcalá de Henares, Madrid), from grapes grown in the vineyard “El Socorro,” belonging to the Instituto Madrileño de Investigación y Desarrollo Rural (IMIDRA) of the Madrid Autonomy. Malvar (MAL) is a traditional white variety of the origin denomination (OD) Vinos de Madrid, while Malvasía (MVS), Viognier (VG), and Chenin Blanc (CHB) are varieties undergoing experimentation and are not authorized in the OD. Interest lies in these varieties as they can complement the characteristics of the traditional varieties, in particular, by increasing the freshness, acidity, and aromatic intensity of the unblended wines. For red varieties, the Tempranillo (TEM) traditional variety of the OD and the authorized Petit Verdot (PV) variety are studied, with the aim of reinforcing the acidity, color intensity, and body of these wines.

Consequently, there is a wide need for developing a simple instrument that can mimic the human sense of smell and be used in routine industrial applications.

## Materials and Methods

### Wine Samples

The wines used in the experiments were elaborated in the IMIDRA with different red and white varieties. The four white varieties, MVS, VG, MAL, and CHB, were made by the traditional method using Vario (Agrovin) as the commercial yeast. The two red varieties, PV and TEM, were made by the traditional method using RVA (Agrovin) as the commercial yeast.

The selection of mixtures of white varieties was done in relation to the aromatic intensity (aromatic profile). The MVS variety is the most aromatic one, followed by CHB and VG varieties (similar aromatic intensity), and finally, MAL is the lowest in aromatic intensity. Thus, MVS was mixed with the other ones to obtain mixture samples that were more differentiated for the analysis. With regards to the red varieties, the native TEM and the authorized PV variety were studied with the aim of reinforcing the acidity, color intensity, and body of the wines. The pairs of wines used are specified in Table 1.

**Table 1:** Composition of the wine mixtures used in this investigation.

Mixture	Wine 1	Wine 2
1	Malvasía (MVS)	Viognier
2	MVS	Malvar
3	MVS	Chenin Blanc
4	Petit Verdot	Tempranillo

The four mixtures were made according to different proportions, in 10% step concentrations (in volume) for the electronic nose (Table 2) and in 25% steps for the human panel (Table 3).

**Table 2:** Wine mixture steps for the electronic nose.

Step	1	2	3	4	5	6	7	8	9	10	11
Wine 1 (%)	0	10	20	30	40	50	60	70	80	90	100
Wine 2 (%)	100	90	80	70	60	50	40	30	20	10	0

**Table 3:** Wine mixture steps for the panel.

Step	1	2	3	4	5
Wine 1 (%)	0	25	50	75	100
Wine 2 (%)	100	75	50	25	0

### Measurement Procedure

The electronic nose and the human nose are very different systems of analysis. Therefore, proper protocol for each system was used to obtain a fair comparison.

#### Electronic Nose Analysis

The measurement system consisted of: the volatile organic compound extraction system; the Peltier cooler; and the WiNOSE 2.0 with a commercial resistive microsensor array. The array consisted of four thin nanocrystalline tin oxide layers deposited over micromechanized silicon hot plates. The sensors worked at temperatures between 200 and 350°C. One of the gas inlets has a carbon filter to provide clean air as a reference baseline. The humidity and temperature sensors, the pump and the flowmeters were located downstream. The details of the setup were described in Aleixandre et al. (2015).

10 ml of each mixture were put into vials and kept at 15°C. To transfer the headspace to the sensors, the flow of air was set at 55 ml/min during the sampling and at 200 ml/min during the recovery. The sampling and recovery times were 1 and 14 min, respectively. Each wine mixture ratio was measured 10 times in succession by the electronic nose. Thus, the measurements were randomized, but all measurements of the same mixture ratio were done together in one batch.

#### Human Panel Analysis

The sensorial analysis to identify the blends of wines of different proportions was carried out by a human olfactory panel of experts. The panel was trained according to the ISO standards related to the methodology (ISO3972:1991), the vocabulary of sensory analysis (ISO3972:1991), the tasting room (ISO8589:1988), and the selection and training of tasters (ISO8586 -1 and -2:1993).

The human panel examined two series of samples: the mixture 1, MVS-VG and the mixture 3, MVS-CHB (Table 1).

To each member of the human panel (8 members), two samples were provided as references: one glass with wine 1 and another glass with wine 2 (Table 1). They separately tasted the wines 1 and 2 for 4 min. Then, they performed three series of quantifications. In each series, three different glasses, with unknown mixture ratios of those two wines, were presented. The possibility that there was more than one mixture in the same proportion was present. Mixtures were classified using Table 4.

**Table 4:** Classification table for the panelists.

100% wine 1	75% wine 1	50% wine 1	25% wine 1	0% wine 1
0% wine 2	25% wine 2	50% wine 2	75% wine 2	100% wine 2

Glass:	Glass:	Glass:	Glass:	Glass:

#### Data Analysis

The measurements of the different wine mixtures were analyzed. The methods of partial least squares (PLS) and artificial neural network (ANN) were used to predict the wine ratios of the mixtures (Wold, 1975; Specht, 1990). Two different validation methods were used (Gutiérrez-Osuna, 2002).

##### Validation 1. Leave One Measurement Out

In this validation method, one measurement is left out and then the algorithms are trained with the rest of the data. In this way, the algorithms predict the concentration of that measurement left out and the prediction is compared with the real wine mixture ratio. The process is repeated for every measurement and then the validation results are compiled and presented.

##### Validation 2. Leave One Mixture Ratio Group Out

In this validation, one mixture ratio group is left out and then the algorithms are trained with the rest of the data. After training, the algorithms predict the concentration of every measurement of the wine mixture of the group left out and then the predictions are compiled and compared with the real wine mixture ratio.

To avoid extrapolation problems with the validation of type 2, the mixture ratios of the 100 and 0% were not included in the validation for both methods.

## Results and Discussion

**Figure 1:**
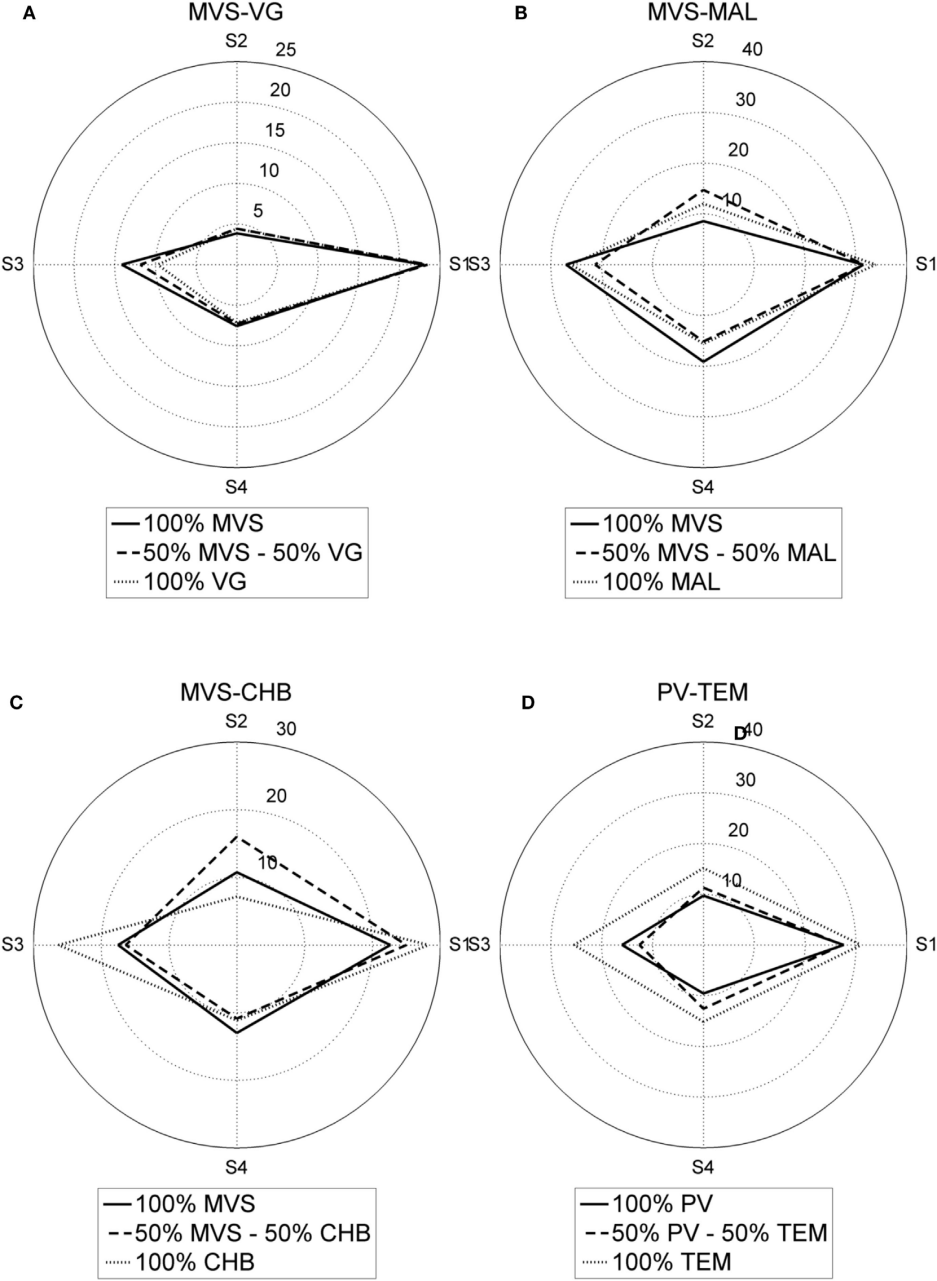
Polar plots of the response of the sensor array for the pure samples and for the mixture 50–50%. **(A)** Malvasía (MVS)-Viognier, **(B)** MVS-Malvar, **(C)** MVS-Chenin Blanc, and **(D)** Petit Verdot-Tempranillo.

Polar plots of the response of the sensor array, for the pure samples and for the mixture 5–50%, are shown in Figure 1, for the four binary mixtures. It is possible to observe a good discrimination.

The root mean square error (RMSE) of the prediction and the regression coefficients (R), for all the mixtures tested by the electronic nose, using the PLS and ANN algorithms, are shown in Tables 5 and 6.

**Table 5:** R values obtained using the electronic nose and the human panel.

Wine mixture	R validation type 1		R validation type 2		R panel
	Partial least squares (PLS)	Artificial neural network (ANN)	PLS	ANN	
Malvasía (MVS)-Viognier	0.791	0.942	0.688	0.910	0.564
MVS-Malvar	0.694	0.887	0.321	0.745	
MVS-Chenin Blanc	0.824	0.964	0.650	0.909	0.367
Petit Verdot-Tempranillo	0.850	0.980	0.716	0.871	

**Table 6:** Root mean square error (RMSE) values obtained using the electronic nose and the human panel.

Mixture	Root mean square error (RMSE) validation type 1 electronic nose		RMSE validation type 2 electronic nose		RMSE panel
	Partial least squares (PLS)	Artificial neural network (ANN)	PLS	ANN	
Malvasía (MVS)-Viognier	16.8	8.7	22.8	12.8	29.5
MVS-Malvar	18.9	11.9	29.4	21.0	
MVS-Chenin Blanc	15.8	7.0	24.9	11.6	37.0
Petit Verdot-Tempranillo	13.9	5.0	20.9	13.0	

As expected, the R coefficients obtained by ANN outperform the ones obtained by PLS for every case (Table 5). In addition, the validation by leaving one mixture ratio group out is stricter than the validation by leaving only one measurement out. This is highlighted in the much lower R coefficient values for the last method. In any case, even for the strictest validation method, there is a clear capability to quantify the mixture ratios for the four wine mixtures: MVS-VG, MVS-MAL, MVS-CHB, and PV-TEM.

The prediction errors obtained show the same trend for the R coefficients as for the two quantification techniques as well as for the two validation methods.

**Figure 2:**
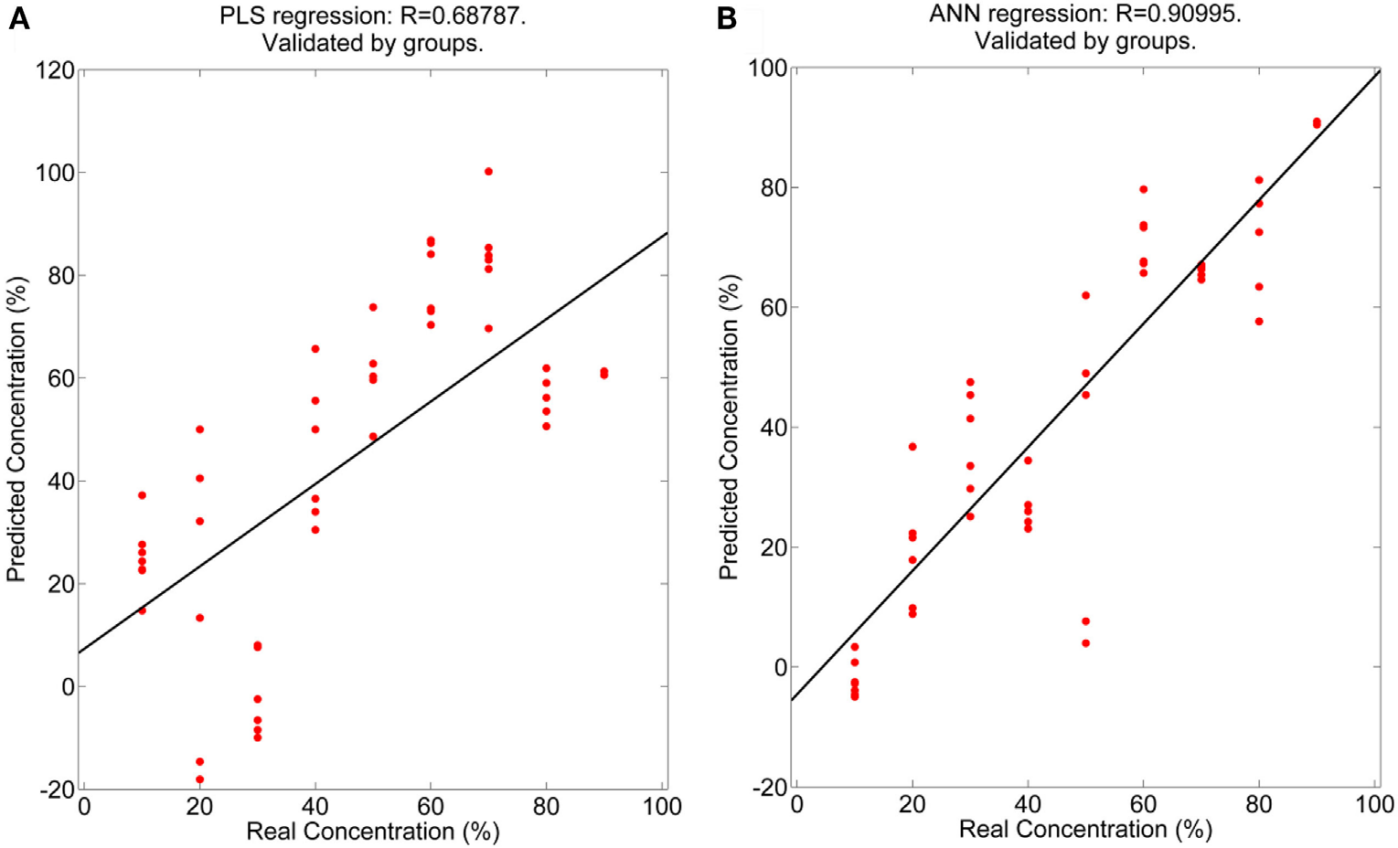
Regression coefficients obtained for the Malvasía (MVS)-Viognier measurements realized with the electronic nose. **(A)** Partial least squares and **(B)** Artificial neural network.

**Figure 3:**
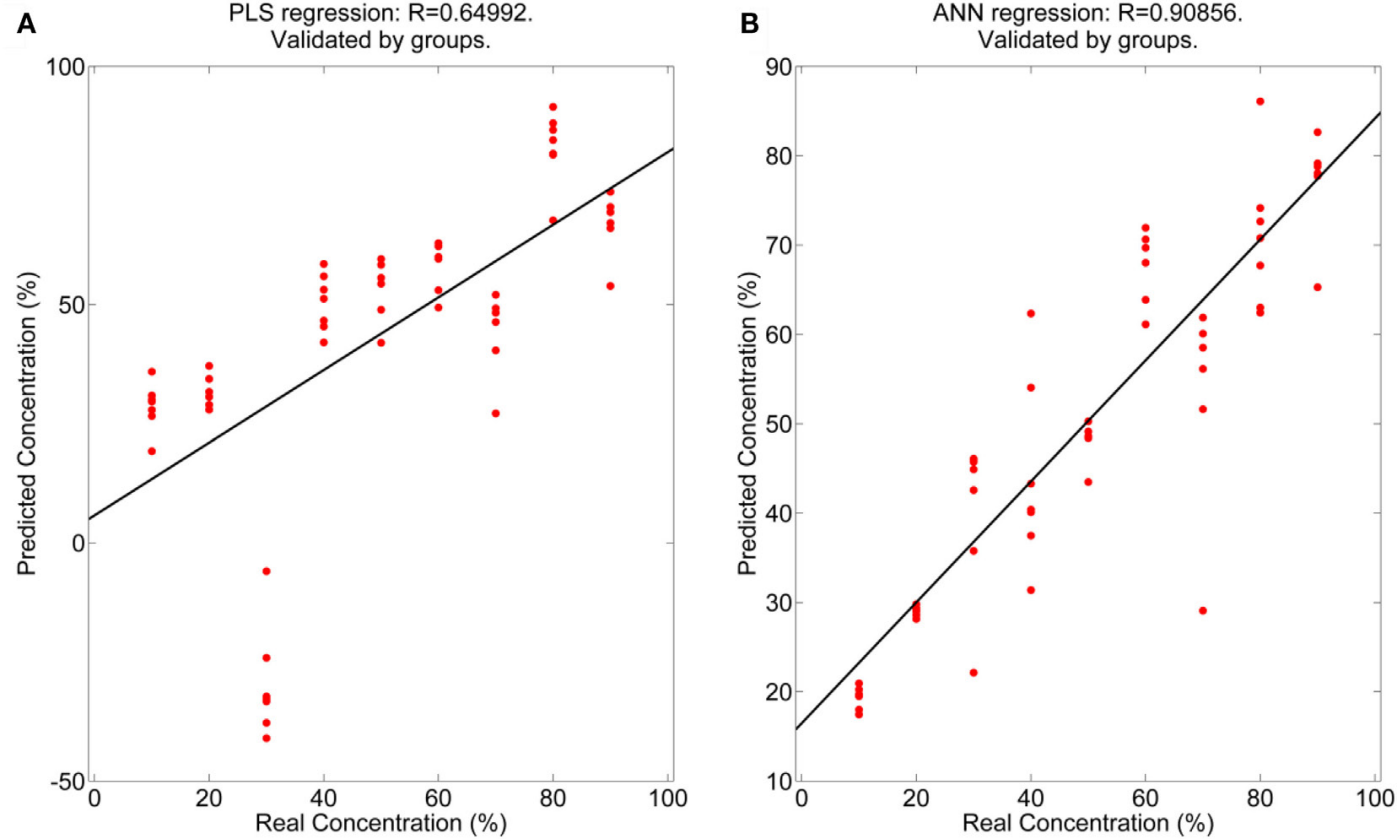
Regression coefficients obtained for the Malvasía (MVS)-Chenin Blanc measurements realized with the electronic nose. **(A)** Partial least squares and **(B)** Artificial neural network.

Only regression graphs, for the mixtures MVS-VG and MVS-CHB, are shown since the best results were obtained with them (Figures 2 and 3). In any case, as mentioned before, all results are given in Tables 5 and 6. This is due to the aromatic profiles being similar between VG and CHB and besides very different with regards to the MVS profile. On the other hand, MAL is a variety with a low aromatic profile and, therefore, more difficult to quantify with the electronic nose.

**Figure 4:**
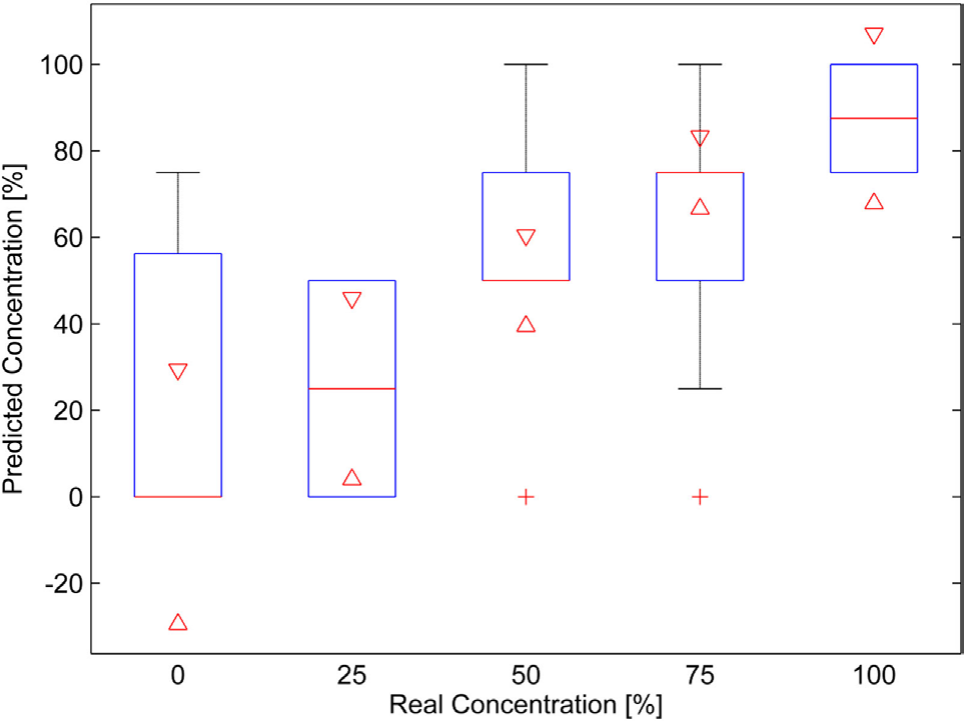
Box plot of the results of the human panel for the Malvasía (MVS)-Viognier mixtures. A 100% concentration corresponds to pure MVS wine.

With respect to the human panel, only the samples MVS-VG and MVS-CHB were analyzed. It is recognized that the human panel does not quantify well the wine mixtures (Atanasova et al., 2005), so for that reason, only the samples that better gave results with the electronic nose were analyzed. The results for the MVS-VG wine mixtures are plotted in the box plot of Figure 4. This graph presents the statistics of the panelist responses in the following way: the red horizontal lines are the medians, the red plus signs are considered outliers, the red triangles are the 95% confidence interval of the medians, the blue boxes are the data between the first and third quartiles, and the black whiskers are the range of data that is not considered an outlier. It can be seen that there is an increasing trend on the medians (red middle line of the boxes) and also on the triangles in the box plots. The intervals overlap for adjacent mixtures, but they do not overlap for distant mixtures, therefore, it can be said, with a 95% confidence, the medians differ for the non-adjacent mixtures.

**Figure 5:**
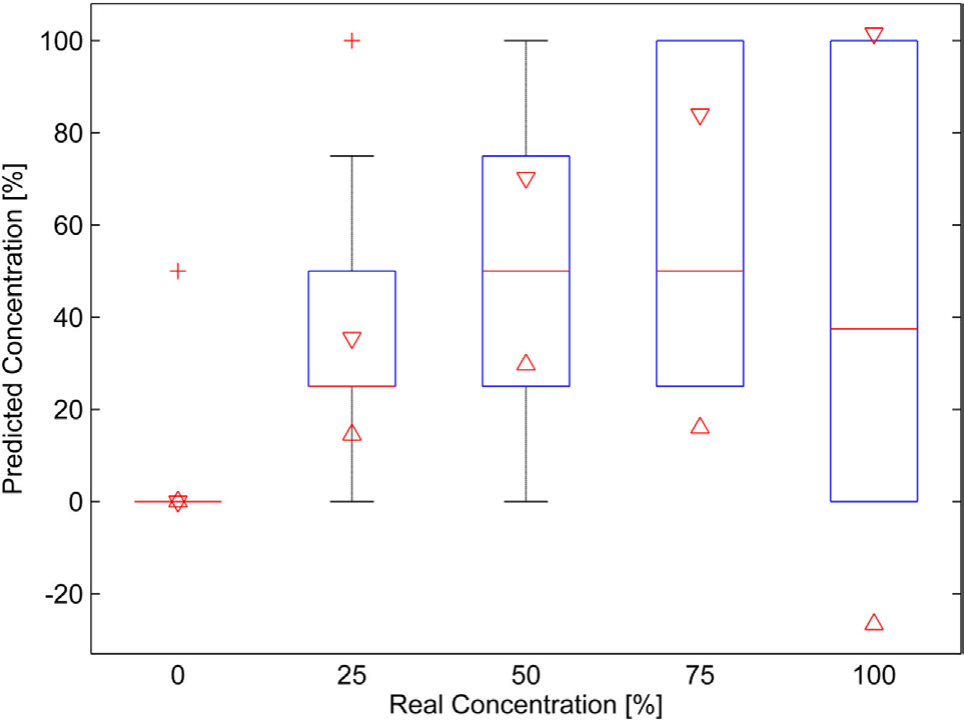
Box plot of the results of the human panel for the Malvasía (MVS)-Chenin Blanc mixtures. A 100% corresponds to pure MVS wine.

The human panel results of the MVS-CHB wine mixtures are presented in Figure 5. The medians (again the red lines) increase also with the concentration, but in this case the panel had more problems quantifying the mixtures and mainly when the concentration increased. This can be seen through the confidence intervals, which decrease with the CHB wine concentration increasing and they overlap for all the mixtures. Therefore, for the human panel, this mixture set is clearly more difficult to quantify than that for the MVS-VG mixture.

**Figure 6:**
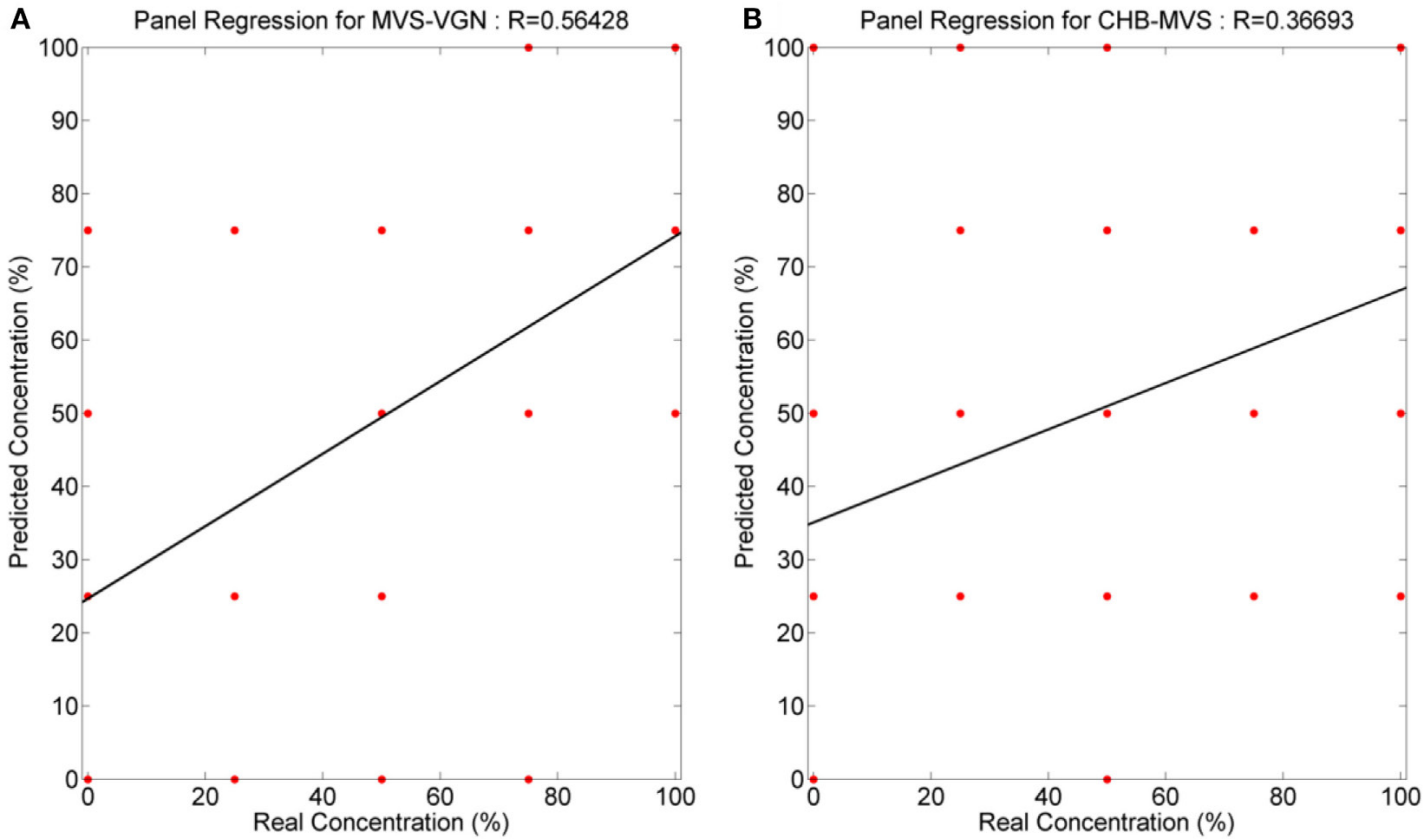
Regression coefficients for the measurements realized with the human panel. **(A)** Malvasía (MVS)/Viognier and **(B)** MVS/Chenin Blanc.

The data of panelist predictions, for each mixture, were used to see if a linear regression could be obtained with respect to real mixture ratio data, as with the data for the electronic nose. In this way, it was possible to compare the quantification achieved using both techniques. Figure 6 shows the regression results. For MVS/VG wine mixtures, the regression coefficient (R) is 0.56 and the RMSE is 29.5 (Figure 6A). For MVS/CHB wine mixtures, the regression coefficient (R) is 0.37 and the RMSE is 37.0 (Figure 6B). These results are also summarized in Tables 5 and 6, and as expected the results were much worse than with those obtained with the electronic nose.

## Conclusion

Wine variety mixtures in different proportions (%) have been quantified with two different technologies: electronic nose and human panel.

For the electronic nose, better results were obtained by applying the ANN than for the PLS for every case, which indicates that the measurement of mixtures is a complex nonlinear problem. In addition, the validation method type 2 is stricter than that for the validation method type 1. It is highlighted by the much lower R coefficient values and the much higher RMSE values. This stricter validation clearly shows the great prediction power of the electronic nose to quantify wine mixture ratios not measured before. In any case, the validation type 2 demonstrates also a clear capability to quantify the mixture ratios for all samples tested.

The best quantification was achieved for the samples of varieties with more intense aromatic profiles.

The human panel encountered difficulties in quantifying the mixture ratios, thus, both the R coefficient and RMSE values were worse than those ones for the electronic nose. There is a consistent rising trend of the quantification as is seen in the box plots. This indicates that the human panel is worse in quantifying the concentration of the mixture ratio than the electronic nose, but it is a good qualitative indicator.

Electronic nose technology has given very good results for performing this wine application, and thus it is important in the enology field.

As a future work, it would be interesting to analyze the influence of the aromatic intensities of other different variety mixtures to get the classification limits of the ANN.

## Author Contributions

MH has directed the research. She has proposed and analyzed the experiments, and has revised the final versión of the manuscript. MA has realized the data treatment and written this part. JC and TA have carried out the experiments related to the panel and written this part. All the authors have discussed the results and have approved the final version of this manuscript.

## Conflict of Interest Statement

The authors declare that the research was conducted in the absence of any commercial or financial relationships that could be construed as a potential conflict of interest.
